# Combination phosphodiesterase type 4 inhibitor and phosphodiesterase type 5 inhibitor treatment reduces non-voiding contraction in a rat model of overactive bladder

**DOI:** 10.1371/journal.pone.0220788

**Published:** 2019-08-28

**Authors:** Brian M. Balog, Abhilasha Tangada, Pooja Sheth, Qi-Xiang Song, Bruna M. Couri, Leah L. Porras, Gary G. Deng, Margot S. Damaser

**Affiliations:** 1 Department of Biomedical Engineering, Lerner Research Institute, the Cleveland Clinic, Cleveland, Ohio, United States of America; 2 Advanced Platform Technology Center, Louis Stokes Cleveland Veterans Affairs Medical Center, Cleveland, Ohio, United States of America; 3 Department of Biology, University of Akron, Akron, Ohio, United States of America; 4 Department of Urology, Changhai Hospital, Naval Medical University, Shanghai, People’s Republic of China; 5 Department of Obstetrics and Gynecology, the Cleveland Clinic, Cleveland, Ohio, United States of America; 6 Lilly Research Laboratories, Eli Lilly and Company, Indianapolis, Indiana, United States of America; 7 Glickman Urological and Kidney Institute, the Cleveland Clinic, Cleveland, Ohio, United States of America; Max Delbruck Centrum fur Molekulare Medizin Berlin Buch, GERMANY

## Abstract

**Introduction:**

Current treatments for overactive bladder (OAB) are often discontinued due to side effects or lack of efficacy. The goal of this study was to determine if combining a phosphodiesterase type 4 inhibitor (PDE4i); with a type 5 inhibitor (PDE5i); would have a beneficial effect on OAB symptoms and if a reduced dose of PDE4i in combination with PDE5i could also provide a beneficial effect in OAB. We hypothesized that PDE5i and PDE4i combination treatment could be utilized to reduce non-voiding contractions and smooth muscle disruption in a rat model of OAB.

**Methods:**

Fifty-eight age-matched Sprague-Dawley rats underwent PBOO and daily gavage with PDE4i alone (roflumilast; 1mg/kg), PDE5i alone (tadalafil;10mg/kg), high dose combination (PDE4i 1mg/kg, PDE5i 10mg/kg), low dose combination (PDE4i 0.2mg/kg, PDE5i 10mg/kg), or vehicle for 28 days. Fourteen animals underwent sham PBOO with vehicle. Rats underwent conscious and anesthetized cystometry 28 days after PBOO and were euthanized for qualitative bladder histology. One-way ANOVA on ranks with a Dunn’s post hoc test was used to indicate statistically significant differences between groups (p<0.05).

**Results:**

Bladder & urethral weight was significantly increased after PBOO with vehicle, PDE4i alone, and PDE5i alone, but not with either combination treatment. Frequency of non-voiding contractions during both conscious and anesthetized cystometry increased significantly after PBOO with vehicle, but not after PDE4i or high dose combination treatments compared to sham PBOO. Threshold pressure for voiding was significantly decreased with high dose combination compared to vehicle. PBOO treated with PDE4i alone or high dose combination showed less bladder smooth muscle fibrosis than vehicle, PDE5i alone, or low dose combination treatments.

**Conclusion:**

A PDE4i and PDE5i combination treatment has potential benefit in reducing OAB symptoms, but future research is needed.

## Introduction

Overactive Bladder (OAB) is defined as urgency, with or without urge incontinence, usually with frequency and nocturia, and affects both men and women at approximately 16% [1,2]. Non-voiding contractions (NVC) are a sign of detrusor overactivity and the primary indicators of OAB, although the causes are not well understood [3]. Current treatments are effective in reducing NVC, however the side effects of anti-muscarinics often lead to discontinuation. The β3-adrenergic receptor agonist, Mirabegron, results in detrusor relaxation from increased cAMP, but has unwanted side effects i.e. increased heart rate and blood pressure [3]. Intravesical injection of onabotulinum toxin A is an alternative treatment but it is invasive, repeated treatments are needed, and it can result in underactive bladder [4,5]. Phosphodiesterase type 5 inhibitors (PDE5i) have also been shown to relieve lower urinary tract symptoms in patients with benign prostatic hyperplasia (BPH) [3].

A number of animal models are used to simulate OAB [6,7]. Hypersensitivity models can be created using different agents, but more closely simulate cystitis than OAB [6]. New models are being created, which could model OAB more closely, but further research is needed on these models before testing new potential therapeutics [8]. The partial bladder outlet obstruction (PBOO) model of OAB in female rats is well validated and demonstrates increased NVC and changes in bladder physiology similar to those seen with OAB in both men and women [6,9,10].

Phosphdiesterases (PDEs) degrade cyclic nucleotides: either cAMP (PDE4) or cGMP (PDE5) or both (PDE3) [4,11,12]. PDE4 and PDE5 are expressed in bladder smooth muscle [13]. PDE4 inhibitors (PDE4i) relax bladder smooth muscle in vitro and could be effective in relieving symptoms of OAB [14]. PDE4i treatments decrease NVC and residual volume in PBOO models [10,11]. However, PDE4 are present in the nervous system and PDE4i in humans can cause nausea and gastrointestinal disruption, so minimizing the dose is important [15]. PDE5i are less effective in treating OAB, but have fewer side effects [3]. The goals of this study were to determine if the combination of a PDE5i and PDE4i would have a beneficial effect on OAB symptoms and if a reduced dose of PDE4i in combination with PDE5i could also provide a beneficial effect in OAB. We hypothesized that PDE5i and PDE4i combination treatment could be utilized to reduce non-voiding contractions and smooth muscle disruption in a rat PBOO model of OAB.

## Materials and methods

All work was approved by the Cleveland Clinic Institutional Animal Care and Use committee (IACUC). Fifty-eight female Sprague-Dawley rats (225-250g) received PBOO and received either: vehicle (2-Hydroxyethyl cellulose solution);(n = 11), PDE4i alone (roflumilast; 1mg/kg);(n = 13), PDE5i alone (tadalafil;10mg/kg);(n = 12), high dose combination treatment (PDE4i 1mg/kg, PDE5i 10mg/kg);(n = 10), or low dose combination treatment (PDE4i 0.2mg/kg, PDE5i 10mg/kg);(n = 12). Fourteen sham PBOO rats received vehicle treatment. Treatments were started the day after PBOO, in a blinded manner. Animals were treated daily via gavage (0.4 ml) for 28 days. Twenty-six days after PBOO or sham PBOO a suprapubic bladder catheter was implanted followed by functional testing and euthanasia 2 days later. Female rats were used because the PBOO model of OAB is the most commonly done and most well validated in female rats [6,10,16,17].

All treatments were prepared at Eli Lilly, labeled in a blinded fashion, and shipped to the Cleveland Clinic. Treatments were stored at 4°C and were continually stirred.

PBOO was performed as previously described [7]. In brief, under isoflurane anesthesia (2%), the abdomen was shaved and a longitudinal mid-line incision was created 1.5 cm above the urethral meatus. The bladder and bladder neck were then isolated. A 1.1 mm rod was placed beside the urethra and 4–0 prolene suture was tied tightly around both the urethra and the rod. The rod was then removed creating, a loose ligature around the urethra, and the ligature was not removed prior to euthanasia. The abdominal muscle and skin were then closed separately. The abdominal incision was closed and Buprenorphine (0.03mg/kg) was given subcutaneously twice daily for two days after surgery as a postoperative analgesic.

Twenty-six days after the PBOO surgery, animals were re-anesthetized for suprapubic bladder catheter implantation, which was done as previously described [18]. An incision was made 1.5 cm above the urethral meatus. After the bladder was isolated, a 4–0 silk purse-string suture was placed in the dome of the bladder. An incision in the center of the purse-string suture was made, through which the catheter (PE-50 tubing with a flared tip) was implanted. The catheter was then tunneled subcutaneously to the back of the animal’s neck and sealed. Bladder size at the time of implant surgery was not recorded.

Twenty-eight days after PBOO, rats were placed in a modified metabolic cage with a beaker on a force transducer (Model FT10; Grass Instruments, West Warwick, RI) placed underneath to measure voided volume. The suprapubic catheter was connected to a syringe pump (Model 200; KD Scientific, New Hope, PA) and a pressure transducer (Model PT300; Grass Instruments). To accommodate the difference in bladder size between sham PBOO and PBOO animals, bladders of sham PBOO animals were filled with saline at 5ml/hr. and bladders of PBOO animals at 7.5 ml/hr. Motion and other artifacts were noted.

Pressure and force data were amplified (Model P122; Grass Instruments) and digitized (10 samples/sec) using a Dash8Xe (AstroMed, West Warwick, RI). Post-void residual volume (PVR) was collected by stopping the flow of saline and disconnecting the catheter from the pump immediately after a void. PVR was collected by gravity filling a pre-weighed falcon tube with urine via the catheter. The tube was weighed and urine was assumed to have the density of water. The first bladder fill allowed the bladder to acclimate to bladder filling and was not included in the analysis. Three filling and voiding cycles with PVR were recorded from each animal. Voided volume, threshold pressure, peak voiding pressure, volume filled, capacity, PVR, duration of voiding cycle, and number of NVC both per void and per minute, were calculated for each voiding cycle. Peak voiding pressure was the highest pressure at the instant of a void. Threshold pressure was the lowest pressure just preceding peak voiding pressure. An NVC consisted of an increase in pressure greater than 20% from baseline, defined as the bladder pressure just prior to a potential NVC presented by an increase in pressure, that did not result in a void, which is similar to other studies in the field (10, 11, 30). Voiding cycle duration started when filling recommenced after PVR collection, and ended with the end of the next void. Volume filled was calculated from the filling rate and the time after PVR collection until the start of a void. Capacity for each void was calculated by adding voided volume and PVR.

After conscious cystometry, rats were anesthetized with urethane intraperitoneally (1.2g/kg) for anesthetized cystometry and the catheter was connected to the urodynamic system with the animals placed supine. Both conscious and anesthetized cystometry were utilized because both have previously been used to assess OAB symptoms [10,19]. Urethane was used as it does not affect bladder reflexes or filling [20]. Three filling and voiding cycles were collected from each animal. Voided volume and PVR were not measured during anesthetized cystometry. Animals were observed and voids were noted. Threshold pressure, peak voiding pressure, volume filled, duration of voiding cycle and number of NVC both per void and per minute, were calculated for each voiding cycle as described above.

Rats were euthanized by CO_2_ immediately after anesthetized cystometry. The lower urinary tract (LUT) consisting of the bladder and urethra were harvested *en bloc*, patted dry, and excess fat was removed. The bladder and urethra were weighed together. The bladder was then filled with one ml of 10% formalin and immersion fixed overnight. Specimen were embedded in paraffin, sectioned (5 μm) and stained with Masson’s trichrome. Histology was qualitatively analyzed in a blinded manner. A few animals that chewed off their catheters before functional testing could not be included in functional outcomes (PBOO with vehicle: 2; PDE5i alone: 2; PDE4i alone: 6; high dose combination: 2; low dose combination: 1), but their organs were harvested and weighed.

Quantitative outcome values calculated for each voiding cycle were averaged to create a mean value for each outcome for each rat, which was then averaged to create a mean and standard error of the mean (SEM) for each group. Each parameter was analyzed independently for outliers, which were removed if they were greater than 2 standard deviations from the mean. A one-way ANOVA on ranks followed by a Dunn’s post hoc test was used to determine differences between all groups with P< 0.05 indicating a statistically significant difference. Quantitative data is presented as mean ± SEM, as recommended by Cumming et al [21]. For the tables the number of animals per group per variable are within the parenthesis. The study was designed with NVC per void during conscious cystometry as the primary outcome and was stopped once it was achieved, resulting in additional outcomes having higher standard error.

## Results

**Fig 1:**
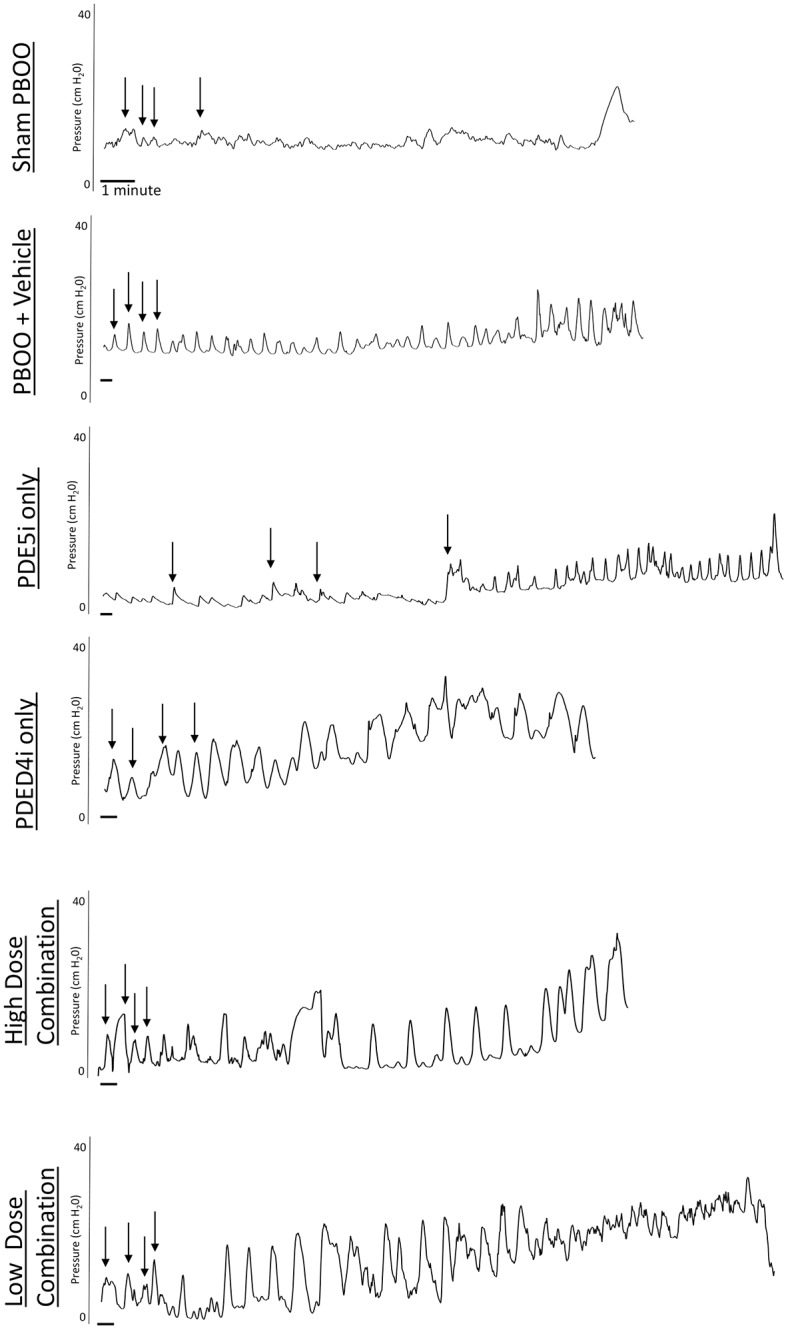
Examples of bladder pressure during conscious cystometry in rats receiving: Sham partial bladder outlet obstruction (PBOO) + vehicle (SPBOO + V) (A), PBOO + vehicle (PBOO + V) (B), PBOO + PDE5i (C), PBOO + PDE4i (D), PBOO + high dose combination treatment (E), and PBOO + low dose combination treatment (F). The scale bar indicates 1 minute. Arrows indicate four example non-voiding contractions.

**Fig 2:**
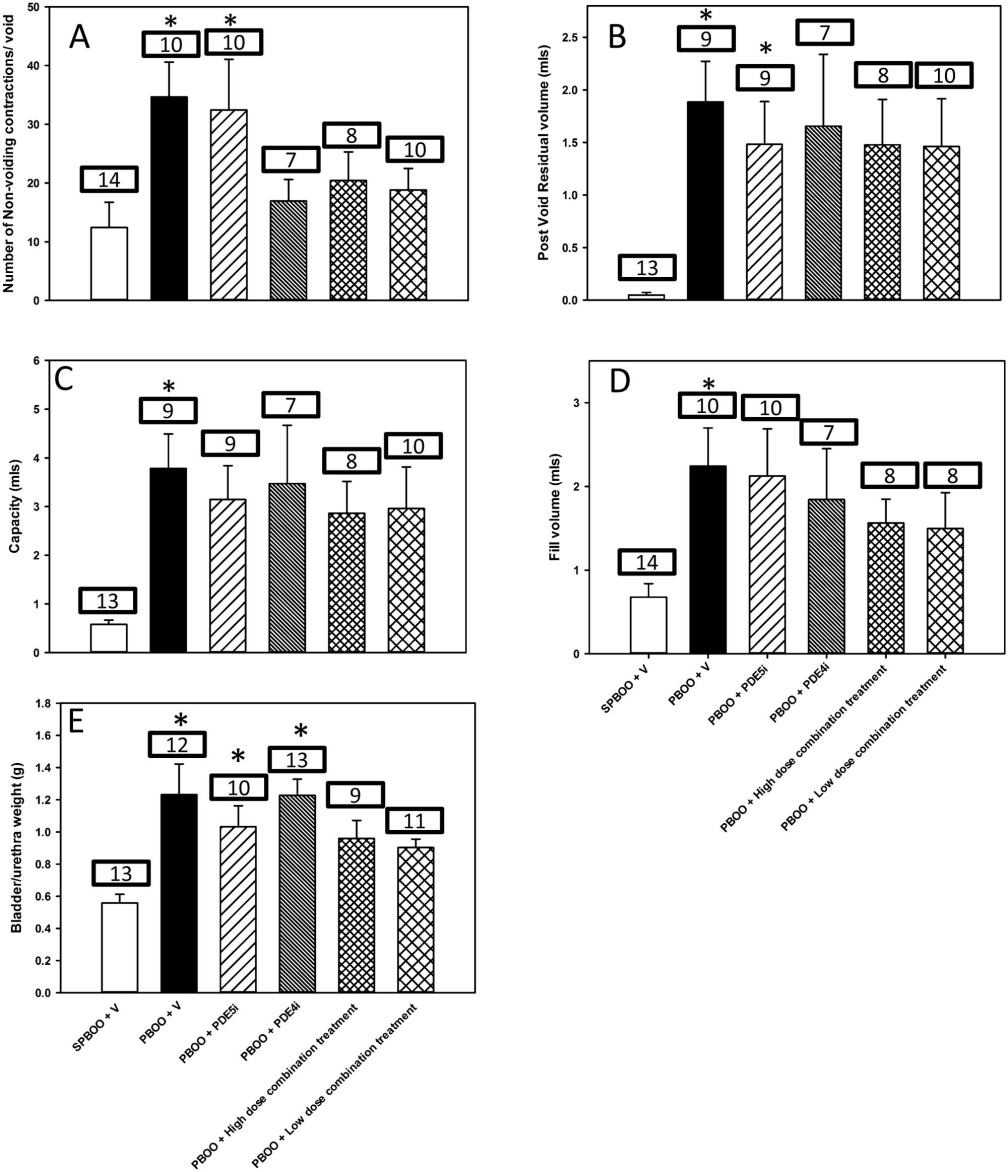
Primary results of conscious cystometry including: Number of non-voiding contractions per void (A), post-void residual volume (B), bladder capacity (C), fill volume (D) and bladder/urethral weights (E) for rats receiving: Sham partial bladder outlet obstruction(PBOO) and vehicle (SPBOO + V), PBOO and vehicle (PBOO + V), PBOO and PDE5i alone (PBOO + PDE5i), PBOO and PDE4i alone (PBOO + PDE4i), PBOO and high dose combination treatment (PBOO + high dose combination treatment) and PBOO and low dose combination treatment (PBOO + low dose combination treatment). Data is presented as mean ± standard error of the mean per group. The number of animals per group per variable are displayed in the box above each bar. *indicates a statistically significant difference compared to SPBOO + V.

The high dose combination group had a morbidity rate of fifty-four percent because of significant weight loss, while the other groups did not have any morbidity. Example conscious cystometry tracings show an increase in number of NVC per void in PBOO + vehicle and PDE5i alone compared to sham PBOO (Fig 1). Examples were chosen based on the number of NVC to best represent the mean per group. There were significantly fewer NVC per void during conscious cystometry in sham PBOO rats (12.4 ± 4.3 /void) compared to those with PBOO receiving vehicle (34.7 ± 5.9 /void) or PDE5i alone (32.5 ± 8.6 /void; Fig 2A). There was no significant difference in number of NVC per void between rats with PBOO receiving PDE4i alone (16.9 ± 3.7 /void), high dose combination (18.8 ± 3.7 /void) or low dose combination (20.4 ± 4.9 /void) treatment compared to sham PBOO animals, indicating decreased OAB in these groups. Nonetheless the frequency of NVC (per minute) was not significantly different between the groups (Table 1).

**Table 1:** Additional outcomes of conscious cystometry.

Animal Group	Non-voiding Contractions per Minute (N)	Voided Volume (ml)(N)	Threshold Pressure (cm H20) (N)	Peak Voiding Pressure (cm H20)(N)
SPBOO + V	1.53 ± 0.29 (14)	0.40 ± 0.07 (14)	12.96 ± 1.07 (14)	26.82 ± 1.64 (14)
PBOO + V	2.22 ± 0.21(10)	0.20 ± 0.04 (10)	15.42 ± 2.26 (10)	31.39 ± 3.87 (10)
PBOO + PDE5i	2.17 ± 0.27 (10)	0.24 ± 0.04 (10)	17.25 ± 2.44 (10)	33.46 ± 3.03 (10)
PBOO + PDE4i	1.23 ± 0.10 (7)	0.39 ± 0.15 (7)	15.57 ± 3.67 (7)	31.27 ± 3.53 (7)
PBOO + high dose combination treatment	1.76 ± 0.23 (8)	0.22 ± 0.04 (8)	15.68 ± 2.16 (8)	28.37 ± 2.42 (8)
PBOO + low dose combination treatment	2.17 ± 0.39 (10)	0.28 ± 0.05 (10)	15.59 ± 2.09 (10)	30.80 ± 2.28 (10)

Sham partial bladder outlet obstruction (PBOO) and vehicle (SPBOO + V), PBOO and vehicle (PBOO + V), PBOO and PDE5i alone (PBOO + PDE5i), PBOO and PDE4i alone (PBOO + PDE4i), PBOO and high dose combination treatment (PBOO + high dose combination treatment) and PBOO and low dose combination treatment (PBOO + low dose combination treatment). The number of animals per group per variable are within the parenthesis.

PVR during conscious cystometry of sham PBOO (0.05 ± 0.02 ml) rats was significantly less than that of rats with PBOO treated with vehicle (1.88 ± 0.38 ml) or PDE5i alone (1.48 ± 0.41 ml; Fig 2B). There was no significant difference in PVR between rats with sham PBOO or PBOO + vehicle or PDE4i alone (1.66 ± 0.68 ml), high dose combination (1.46 ± 0.45 ml), or low dose combination (1.48 ± 0.43ml) treatment. Bladder capacity of sham PBOO rats was 0.58 ± 0.31ml, which was significantly less than rats with PBOO and vehicle (3.78 ± 0.71 ml; Fig 2C). However, bladder capacity was not significantly different between sham PBOO and PBOO with PDE4i alone (3.47 ± 1.20 ml) or PDE5i alone (3.14 ± 0.70 ml), high dose combination (2.86 ± 0.65 ml), or low dose combination (2.96 ± 0.85 ml) treatment.

Average volume filled during conscious cystometry in the PBOO and vehicle group (2.24 ± 0.5 ml) was significantly greater than that of sham PBOO rats (0.67 ± 0.6 ml; Fig 2D). No other groups were significantly different from one another.

Bladder and urethral weight of sham PBOO rats was 0.56 ± 0.05 g, significantly less than that of rats with PBOO receiving vehicle (1.23 ± 0.19g), PDE4i alone (1.22 ± 0.10g) or PDE5i alone (1.03 ± 0.13g) (Fig 2E). There were no significant differences between LUT weight of rats with PBOO and vehicle and those of with PBOO receiving PDE4i alone or PDE5i alone. LUT weight of rats with PBOO and both combination treatments was not significantly different from that of either sham PBOO or PBOO with vehicle.

**Fig 3:**
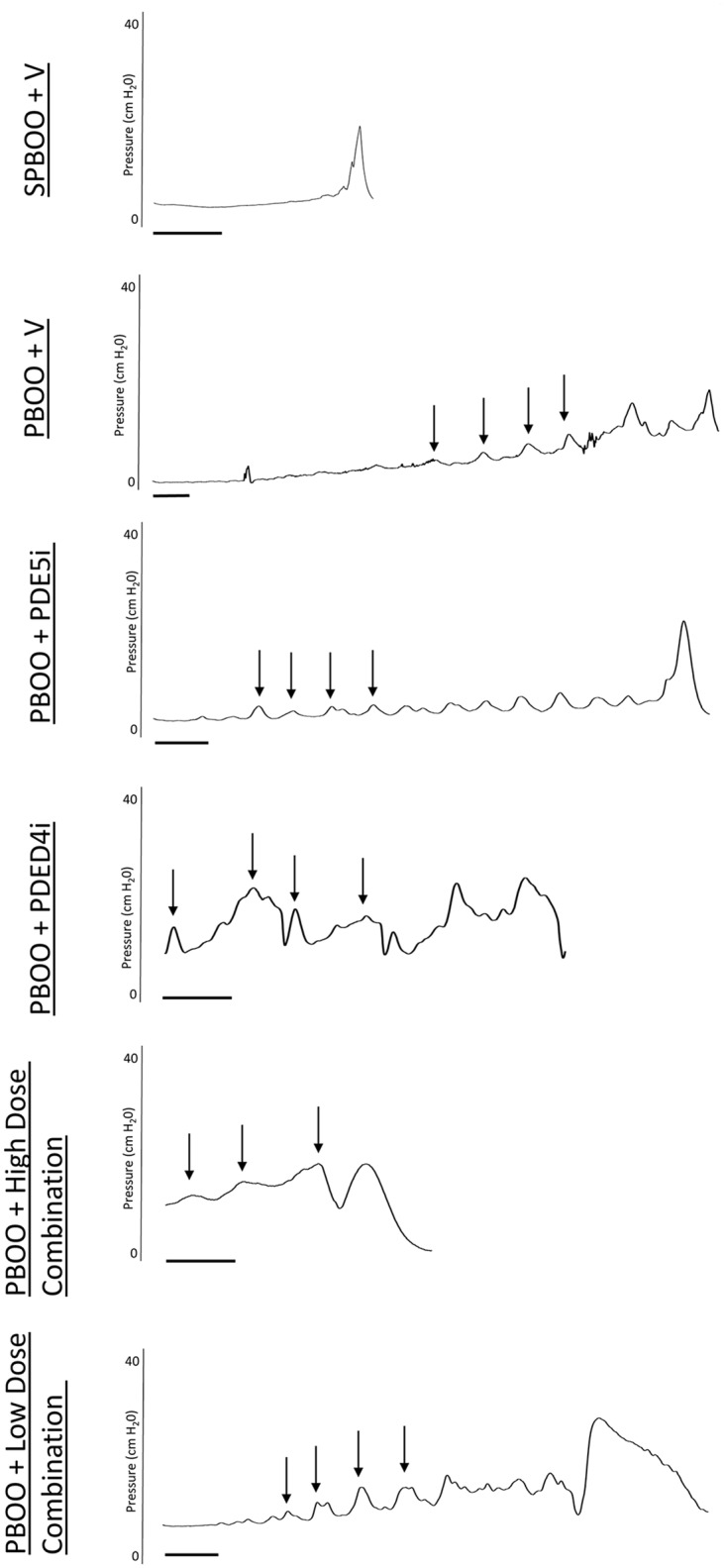
Examples of anesthetized cytometry bladder pressure for each group: Sham partial bladder outlet obstruction (PBOO) and vehicle treatment (SPBOO + V) (A), PBOO with vehicle (PBOO + V) (B), PBOO with PDE5i treatment (C), PBOO with PDE4i treatment (D), PBOO with high dose combination treatment (E), and PBOO with low dose combination treatment (F). The scale bar indicates 1 minute. Arrows indicate example non-voiding contractions.

**Fig 4:**
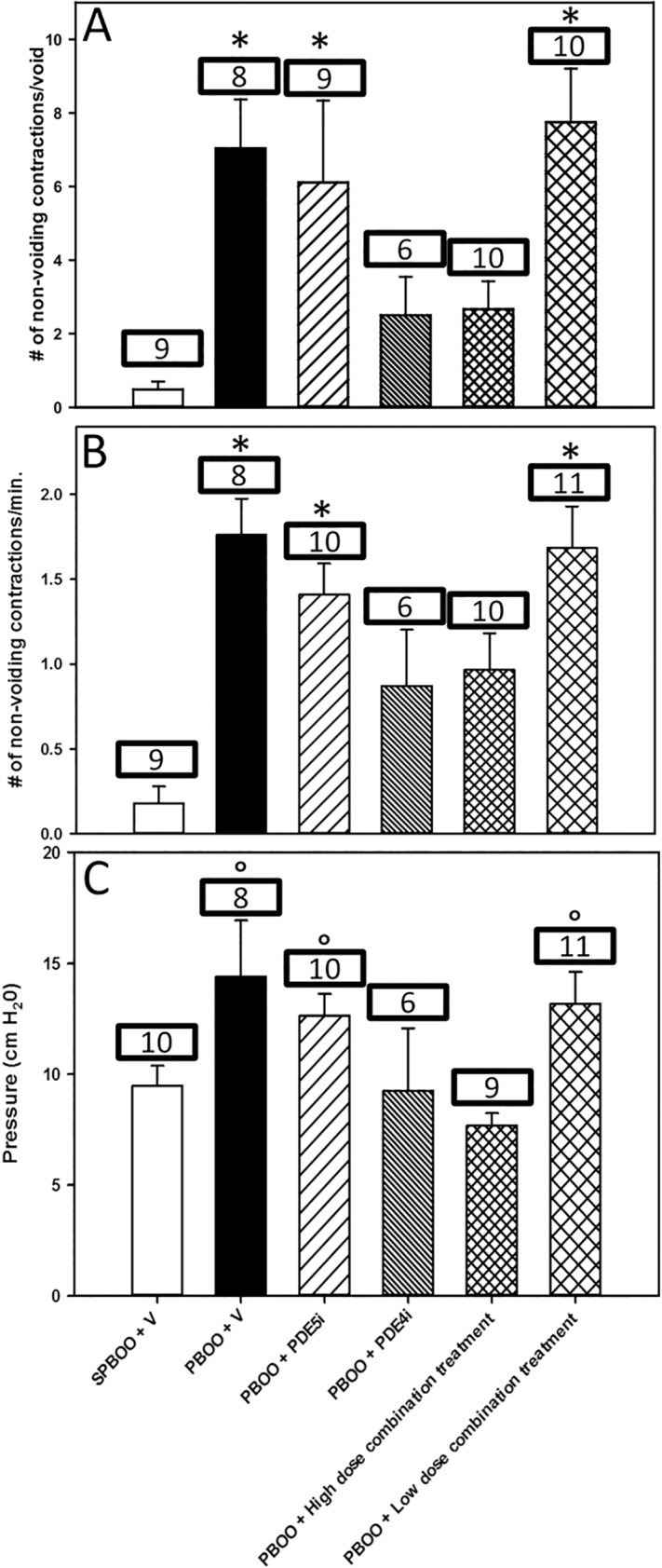
Primary results of anesthetized cystometry including: Number of non-voiding contraction per void (A), Non-voiding contraction per minute (B), and threshold voiding pressure (C) in rats receiving: Sham partial bladder outlet obstruction (PBOO) and vehicle (SPBOO +V), PBOO and vehicle (PBOO + V), PBOO and PDE5i alone (PBOO + PDE5i), PBOO and PDE4i alone (PBOO + PDE4i), PBOO and high dose combination treatment (PBOO + high dose combination treatment) and PBOO and low dose combination treatment (PBOO + low dose combination treatment). Data is presented as mean ± standard error in each group. The number of animals per group per variable are displayed in the box above each bar. *indicates a statistically significant difference compared to SPBOO + V; ° indicates a statistically significant difference compared to PDE4i high dose combination treatment.

Example anesthetized cystometry data (Fig 3) shows an increase in NVC in PBOO with vehicle, PBOO with PDE5i alone and PBOO with the low dose combination treatment groups compared to sham PBOO. Examples were chosen based on the number of NVC to best represent the mean per group. The number of NVC per void during anesthetized cystometry in rats with sham PBOO (0.48 ± 0.2 /void) was significantly less than that of rats with PBOO receiving vehicle (7.04 ± 1.3 /void), PDE5i alone (6.11 ± 2.3 /void), or low dose combination (7.7 ± 1.5 /void) treatments (Fig 4A). There were no significant differences between sham PBOO and PBOO with PDE4i alone (2.5 ± 1.0 /void) or high dose combination (2.6 ± 0.7 /void) treatments.

The number of NVC per minute during anesthetized cystometry was significantly increased in PBOO animals treated with vehicle (1.8 ±. 0.6/min), PDE5i alone (1.4 ± 0.2 /min) or low dose combination treatment (1.7 ± 0.2 /min) compared to sham PBOO (0.2 ± 0.1 /min; Fig 4B). There were no significant differences in number of NVC per minute between sham PBOO and PBOO with PDE4i alone (0.9 ± 0.3 /min) or high dose combination (1.0 ± 0.2 /min) treatment.

There was a significant decrease in threshold pressure during anesthetized cystometry in rats with PBOO receiving high dose combination treatment (7.7 ±. 0.6 cm H_2_0) compared to PBOO with vehicle (14.4 ±. 2.5 cm H_2_0), PDE5i alone (12.6 ±. 1.0 cm H_2_0) and low dose combination (13.2 ±. 1.4 cm H_2_0) treatment (Fig 4C). Threshold pressures of rats with PBOO receiving PDE4i alone (9.2 ±. 2.8 cm H_2_0) or high dose combination treatments were not significantly different compared to rats with sham PBOO (9.5 ±. 0.9 cm H_2_0). Other outcomes were not significantly different between the groups (Table 2).

**Table 2:** Additional outcomes of anesthetized cystometry.

Animal Group	Fill volume (ml) (N)	Peak Voiding Pressure (N)
SPBOO + V	0.43 ± 0.08 (10)	24.04 ± 2.60 (10)
PBOO + V	0.33 ± 0.07 (8)	24.51 ± 3.77 (8)
PBOO + PDE5i	0.48 ± 0.08 (10)	20.96 ± 1.90 (10)
PBOO + PDE4i	0.41 ± 0.07 (6)	17.77 ± 2.36 (6)
PBOO + high dose combination treatment	0.31 ± 0.05 (10)	21.74 ± 4.44 (10)
PBOO + low dose combination treatment	0.68 ± 0.13 (11)	22.82 ± 2.14 (11)

Sham partial bladder outlet obstruction (PBOO) and vehicle (SPBOO + V), PBOO and vehicle (PBOO + V), PBOO and PDE5i alone (PBOO + PDE5i), PBOO and PDE4i alone (PBOO + PDE4i), PBOO and high dose combination treatment (PBOO + high dose combination treatment) and PBOO and low dose combination treatment (PBOO + low dose combination treatment). The number of animals per group per variable are within the parenthesis.

**Fig 5:**
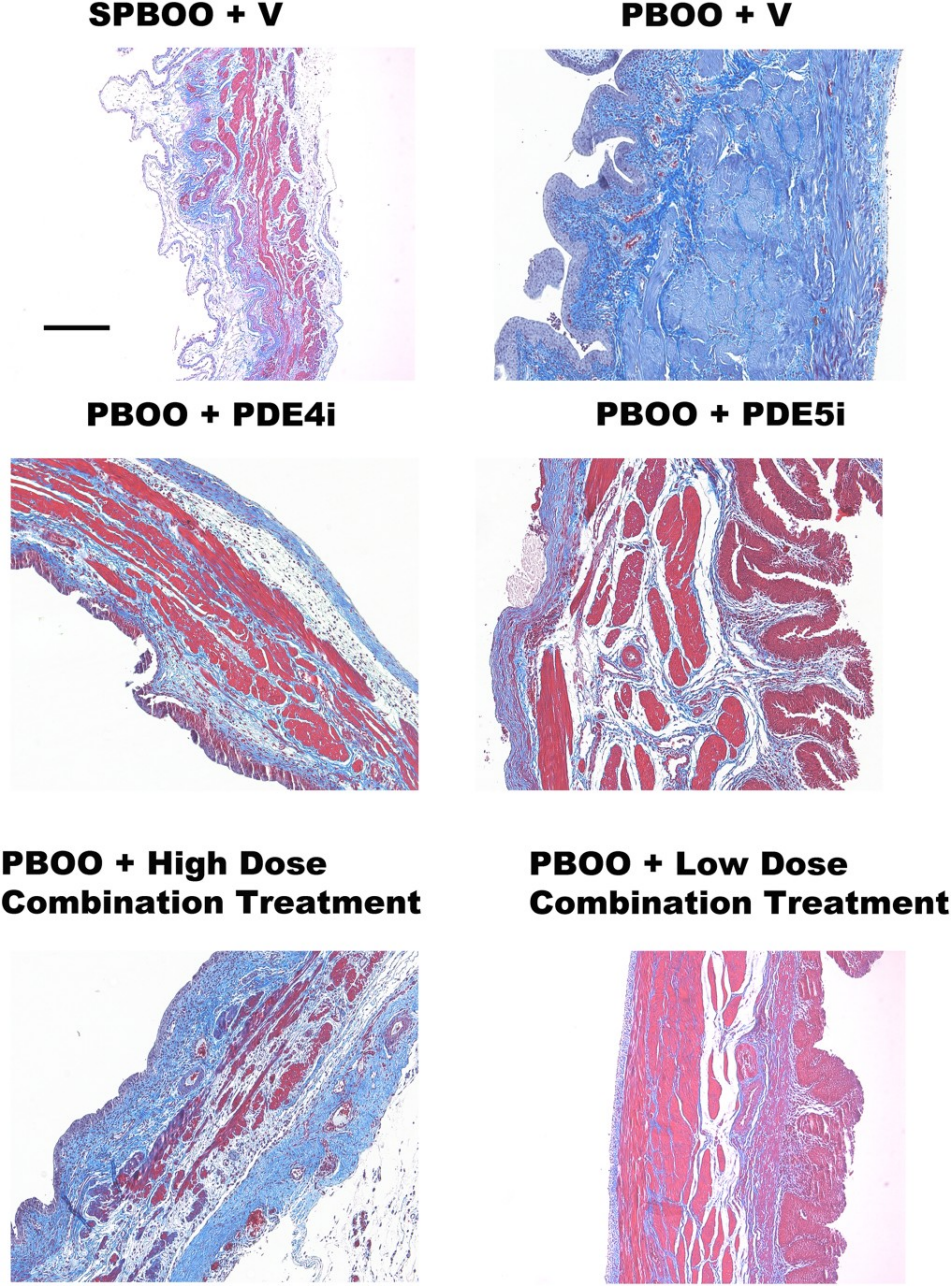
Examples of Masson’s trichrome-stained transverse sections of bladder from animals in all experimental groups: Sham partial bladder outlet obstruction (PBOO) and vehicle (SPBOO + V), PBOO and vehicle (PBOO + V), PBOO and PDE5i alone (PBOO + PDE5i), PBOO and PDE4i alone (PBOO + PDE4i), PBOO and high dose combination treatment (PBOO + high dose combination treatment) and PBOO and low dose combination treatment (PBOO + low dose combination treatment). Scale Bar is 100 μm in length. Collagen signifying fibrosis is stained blue, muscle is stained red.

Bladder wall thickness increased after PBOO in all groups compared to sham PBOO + (Fig 5). Less fibrosis was observed in the bladders of PBOO animals treated with PDE4i alone or high dose combination compared to PBOO rats with sham treatment, PDE5i alone, or low dose combination.

## Discussion

PDE5i can be used to treat symptoms of bladder outlet obstruction in patients with BPH [22]. PDE5i have been shown to increase nitric oxide (NO) signaling and bladder relaxation in a rat PBOO model [23]. In addition, PDE4i have been shown to relax bladder smooth muscle in vitro and decrease NVC and residual volume in PBOO models [10–12,24]. Strips of human bladder muscle had greater relaxation with intact urothelium than without urothelium when treated with PDE4i, suggesting that since NO is produced in the urothelium, NO signaling could enhance the effects of PDE4i treatment [13,14]. However, PDE4i treatment has significant side effects. Both cAMP and cGMP can cause relaxation of smooth muscle through activation of protein kinase A (PKA) (cAMP) and protein kinase G (PKG) (cGMP) [25]. The combination of both PDE4i and PDE5i could potentially be given orally and provide symptomatic relief at a lower dosage than either one given individually. The aims of the study were to determine if there are beneficial effects on OAB symptoms with combination PDE4i and PDE5i and if those effects could be achieved with a reduced PDE4i dose combined with PDE5i. We hypothesized that PDE5i and PDE4i combination treatment could be utilized to reduce non-voiding contractions and smooth muscle disruption in a rat PBOO model of OAB.

The doses chosen for this study were selected based on previous publications using PDE4i and PDE5i [11,26]. Clinically, 10 mg of tadalafil has been shown to be safe and effective, while 10mg/kg has previously been used to investigate the effects of tadalafil in rodent experiments [26–28]. Previous investigations of PDE4i in rodent PBOO models have used a range of doses between 0.1mg/kg– 1mg/kg and roflumilast has been shown to be safe in rodents [10,11,29].

We demonstrated an increase in the number of NVC per void in conscious cystometry after PBOO, demonstrating consistency of the model [30]. PDE5i alone did not decrease the frequency of NVC, while treatments containing PDE4i restored NVC to those of sham PBOO animals, consistent with previous reports that have shown PDE4i treatment can reduce the number of NVC [10,11]. Nishiguchi et al. showed that PDE4i can reduce NVC at doses as low as 0.1 mg/kg when injected before cystometry [10]. Similarly, our results demonstrate that PDE4i reduces NVC. However, we did not test our PDE4i low dose (0.2 mg/kg) alone, which would have determined if the reduction detected in the low dose combination was because of a decreased dose of PDE4i or the combination of PDE4i and PDE5i, although the results of the Nishiguchi et al. study suggest we would have seen a decrease in NVC. The purpose of the study was to determine if the combination treatment would have a functional effect. A future study to determine the mechanism of action would include a PDE4i low dose alone group. Neither the low or high dose combination groups were significantly different from either the Sham PBOO or PBOO with vehicle in conscious cystometry outcomes, indicating that the combination treatments partially recovered bladder function.

Anesthetized cystometry showed a significant increase in NVC both per min and per void in PBOO with vehicle compared to sham PBOO, again demonstrating consistency of the model [31]. PDE5i alone and the low dose combination treatment were not effective in reducing NVC per minute or per void, demonstrating that PDE4i alone and the high dose combination treatment were more effective in reducing NVC during anesthetized cystometry in comparison to conscious cystometry, possibly due to reduced motion artifact during anesthetized cystometry enabling greater precision of measurement. Our PDE4i results are similar to the effects seen in a cystitis model with increased NVC, in which intravesical PDE4i decreased the number of NVC [32]. Likewise, we observed a significant decrease in threshold pressure during anesthetized cystometry in rats treated with the high dose combination treatment compared to PBOO with vehicle, PDE5i alone or low dose combination treatments. Similarly, intravesical PDE4i treatment resulted in decreased threshold pressure during anesthetized cystometry in the cystitis model [32]. Since PDE4i was administrated intravesically in the cystitis study, a dose comparison with our study is not possible [32].

PVR measured during conscious cystometry was increased after PBOO treated with vehicle or PDE5i alone compared to sham PBOO. In contrast when PBOO was treated with PDE4i alone, low or high dose combination treatments PVR was not significantly different from that of Sham PBOO or PBOO with vehicle. In Nishiguchi’s study PDE4i demonstrated a significant decrease in PVR at 1mg/kg, but there was not a significant decrease at lower doses: 0.1 or 0.5 mg/kg, suggesting our low dose (0.2mg) would not have an effect on PVR either; however it is important to mention that in the Nishiguchi study PDE4i was administered via IV injection at the time of cytometry [10]. While previous studies have not reported the effects of PDE5i on PVR after PBOO, in our study the addition of a low dose (0.2mg/kg) of PDE4i to PDE5i, improved the effectiveness of the PDE5i dose at reducing PVR.

Similar to previous studies, we demonstrated a significant increase in LUT weight after PBOO [31]. Treatment with either PDE5i or PDE4i alone after PBOO did not decrease LUT weight indicating that neither PDEi alone reduced the increase in bladder size due to the PBOO, consistent with Kang et al., who showed that Mirodenafil, a PDE5i, did not significantly decrease bladder weight after PBOO [33]. Similarly, Matsumoto et al. showed that a different PDE5i, Vardenafil, did not significantly reduce bladder weight after PBOO. However, Kawai et al. showed that Tadalafil, a PDE5i, significantly reduced bladder size after PBOO [16,34]. These results indicate more research is needed to determine which PDE5i is more effective in reducing bladder weight. Studies investigating PDE4i and PBOO did not report bladder weight.

In our study, LUT weight after PBOO and either low or high dose combination treatment was not significantly different from sham PBOO, indicating that combination treatment could potentially lessen the effects of PBOO. Matsumoto et al. tested cilostazol, a PDE3i, which did not significantly reduce bladder weight after four weeks of treatment [17]. It is unclear if the combination of PDE4i, which increases cAMP, and a PDE5i, which increases cGMP, would have a similar effect as a PDE3i, which increase cAMP and cGMP. Dosing in our study may have been higher since Cilostazol was mixed in the food and exact mg/kg dosing was not stated in the Matsumoto et al. study [17].

An increase in LUT weight was supported by an increase in bladder capacity and fill volume after PBOO with vehicle treatment, but not with any of the PDEi treatments. LUT weight, capacity and fill volume were secondary measures and our project was not powered to show a significant difference; however we did show a difference between sham PBOO and PBOO with vehicle, similar to prior studies [35]. Beamon et al did not show a decrease in capacity with a PDE5i, supporting our findings [35]. It is important to note that the filling rates used in this study are not physiological filling rates and that, while different filling rates for the Sham PBOO and PBOO groups have previously been utilized, it could be a limitation. Previous work by Lluel et al., did not see changes in bladder function when increasing flow rate in PBOO animals, suggesting it would not have affected the results in this study [36].

Masson’s trichrome staining demonstrated less fibrosis with PDE4i alone or high dose combination treatment after PBOO compared to PBOO with vehicle. In addition, there was an increase in fibrosis in PBOO with vehicle compared to sham PBOO, with the PDE5i alone group being comparable to PBOO and vehicle. This is in contrast to Beamon et al. who reported that PDE5i reduced fibrosis [35]. The difference of these results could be explained by the difference in length of treatment time between the studies: 6 weeks in Beamon et al. and 4 weeks in our study. Had we utilized a longer treatment time in our study we may have observed similar results.

A limitation of our study design was not including a low dose PDE4i treatment group, since Nishiguchi et al. demonstrated PDE4i decreased NVC at 0.1mg/kg [10]. The inclusion of a low dose PDE4i treatment group may have allowed us to determine if the effects of the low dose combination treatment group were due to synergistic effects of the combination treatment. Our study was intended to determine any benefits of a PDE4i and PDE5i combination treatment on voiding function after PBOO. Additionally, the study was designed with NVC per void during conscious cystometry as the primary outcome and was stopped once it was achieved, resulting in additional outcomes having higher standard error. Additionally, animals in the high combination group had increased morbidity as many lost weight, animals that lost more than 10% of their initial body weight were removed from the study early. An additional limitation to the study is that we did not quantitatively analyze collagen content in the histological sections. Future studies are needed to investigate the mechanism of action of the combination treatment.

## Conclusion

Both combination treatments were more effective in improving bladder function than PDE4i, PDE5i alone or vehicle. Conscious cystometry demonstrated both combination treatments had similar effects, while the high dose combination treatment was more effective than the low dose combination treatment at reducing NVC in anesthetized cystometry; however the high dose combination had a higher morbidity rate. Therefore, a lower PDE4i dose may be more effective with a higher PDE5i dose, but further testing is needed.
